# The Interactions of Nintedanib and Oral Anticoagulants—Molecular Mechanisms and Clinical Implications

**DOI:** 10.3390/ijms22010282

**Published:** 2020-12-30

**Authors:** Grzegorz Grześk, Anita Woźniak-Wiśniewska, Jan Błażejewski, Bartosz Górny, Łukasz Wołowiec, Daniel Rogowicz, Alicja Nowaczyk

**Affiliations:** 1 Department of Cardiology and Clinical Pharmacology, Faculty of Health Sciences, Ludwik Rydygier Collegium Medicum in Bydgoszcz, Nicolaus Copernicus University in Toruń, 87-100 Toruń, Poland; g.grzesk@cm.umk.pl (G.G.); atww@interia.pl (A.W.-W.); janbi@o2.pl (J.B.); gornyb@wp.pl (B.G.); lordtor111@gmail.com (Ł.W.); rogowicz.d@gmail.com (D.R.); 2 Department of Organic Chemistry, Faculty of Pharmacy, Ludwik Rydygier Collegium Medicum in Bydgoszcz, Nicolaus Copernicus University in Toruń, 2 dr. A. Jurasza St., 85-094 Bydgoszcz, Poland

**Keywords:** nintedanib, idiopathic pulmonary fibrosis, direct oral anticoagulants

## Abstract

Nintedanib is a synthetic orally active tyrosine kinase inhibitor, whose main action is to inhibit the receptors of the platelet-derived growth factor, fibroblast growth factor and vascular endothelial growth factor families. The drug also affects other kinases, including Src, Flt-3, LCK, LYN. Nintedanib is used in the treatment of idiopathic pulmonary fibrosis, chronic fibrosing interstitial lung diseases and lung cancer. The mechanism of action suggests that nintedanib should be considered one of the potential agents for inhibiting and revising the fibrosis process related to COVID-19 infections. Due to the known induction of coagulation pathways during COVID-19 infections, possible interaction between nintedanib and anticoagulant seems to be an extremely important issue. In theory, nintedanib could increase the bleeding risk, thrombosis and lead to thrombocytopenia. The data from clinical trials on the concomitant use of nintedanib and antithrombotic agents is very limited as this patient group was within the standard exclusion criteria. Nintedanib is an important therapeutic option, despite its interaction with anticoagulants. If anticoagulant therapy is necessary, the more effective and safer option is the concomitant administration of DOACs and nintedanib, especially when drug-monitored therapy will be used in patients at high risk of bleeding complications.

## 1. Introduction

**Figure 1:**
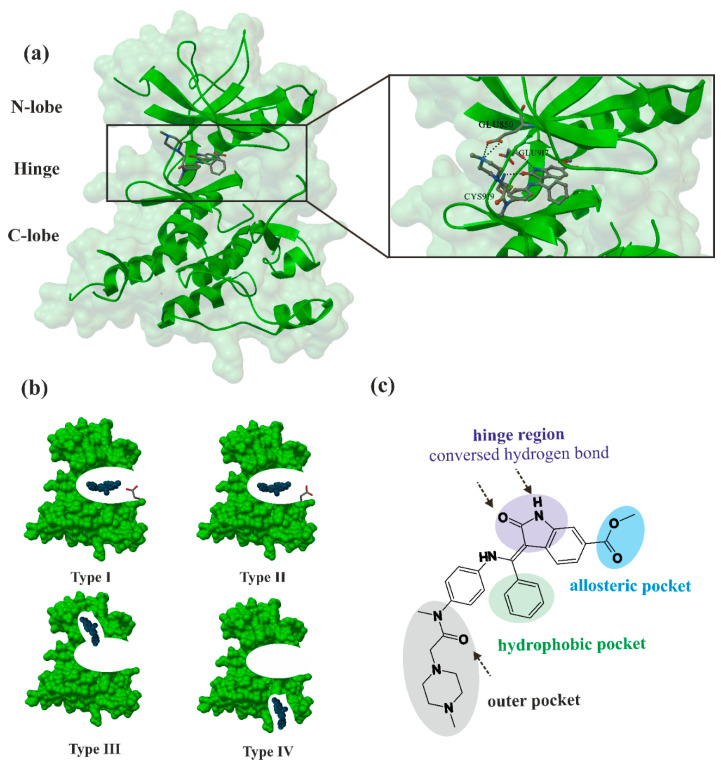
Transmembrane receptor RTK, different types of reversible inhibition and graphical representation of nintedanib RTK pharmacophore features. (**a**) X-ray structure of nintedanib bond in the active site of the VEGFR-2 crystal with the enlarged area showing the structural elements around the ligand-binding site (PDB ID: 3C7Q, 2.10 Å) [21]. The molecular mechanism of action of nintedanib consists of the competitive binding to the adenosine triphosphate (ATP) pocket. The result is inhibition of intracellular signaling vital for the proliferation, migration, and transformation of fibroblasts, which are fundamental to IPF [22]. (**b**) The literature-derived classification of reversible inhibitor types: where type I is binding to the active kinase hinge area (DFG-in) while type II is binding to the respective inactive area (DGF-out). Both inhibitor types compete directly with ATP by stabilizing enzymatically relevant kinase conformations. Inhibitors of type III and IV allosterically interact outside the hinge area (i.e., in its close vicinity or back) or activate remote residues in the kinase domain [14,23]. (**c**) Pharmacophore points defined according to the literature for nintedanib structure [1,14].

Nintedanib is an unusual molecule that does not occur in nature. It is a purely synthetic compound obtained in 1998, during a lead optimization program for small-molecule inhibitors of angiogenesis [1]. From the chemical point of view nintedanib (development code BIBF 1120) is a small molecule (MW = 539,6248 g/mol), ethanesulfonate diastereomer of methyl (3Z)-3-{[(4-{methyl[(4-methylpiperazin-1-yl)acetyl]amino}phenyl)amino](phenyl)methylidene}-2-oxo-2,3-dihydro-1H-indole-6-carboxylate. It is a derivative of indolinone, a secondary amine composed of a benzene and pyrrole ring. It is highly crystalline (mp = 305 °C) and exhibits a lipophilic character (log P ≈ 3.0) and good aqueous solubility (>20 mg/mL in water) [2]. The chemical structure is shown in Figure 1.

Nintedanib (ATC code: L01XE31 [3]) is an orally active tyrosine kinase inhibitor. It has been evaluated in large clinical trials for the treatment of idiopathic pulmonary fibrosis (IPF) (ICD-10-CM code: J84.112 [4]), a fatal disease leading to progressive loss of lung function. This disease involves uncontrolled proliferation of lung fibroblasts and excessive deposition of extracellular matrix (ECM) by myofibroblasts [5,6]. Six years ago, nintedanib was globally approved as the first drug in the treatment of IPF [7,8,9]. In the EU it was approved for the treatment of lung cancer in 2014 [10,11]. In March 2020, the FDA extended its use in the US to treat chronic fibrosing interstitial lung diseases (ILD) with a progressive phenotype (trait) [12]. It should be emphasized here that nintedanib is the first targeted chemotherapy in patients with IPF and currently one of only two drugs available for IPF [2]! Molecularly targeted cancer therapy involves a selected class of drugs which play an important role in the carcinogenicity or proliferation of the tumor. The mechanism of their action consists of inhibition of growth and spread of cancer. This is done by interfering with specific molecules critical to tumor progression, for example a specific tyrosine kinase agent. Currently available evidence suggests that the molecularly targeted cancer therapy therapeutic effects are comparable or in some cases higher with respect to the effects of classical methods, i.e., chemotherapy and radiotherapy. It seems reasonable to suggest, at this point, that molecularly targeted therapy is safer for normal cells.

In this paper the most important information concerning the mechanism of action and adverse reactions of nintedanib has been collected. Particularly, attention is paid to the recent topics related to the interaction of nintedanib and selected direct oral anticoagulants (DOACs). DOACs according to the current evidence seem to be a better and safer therapeutic option for patients with clinical indications to both therapies.

## 2. Receptor Tyrosine Kinases (Phosphotransferases)

**Scheme 1:**
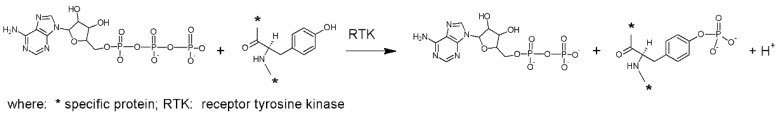
Phosphorylation reaction of phosphate moieties transfer from ATP to tyrosine residues of specific proteins [17].

Receptor tyrosine kinases (RTK) belong to a family of transmembrane receptors for extracellular signaling molecules. The family includes growth factors and hormones [13]. They are enzymes catalyzing the transfer of the γ-phosphate group of adenosine triphosphate (ATP) to the hydroxyl group of tyrosine residues of specific proteins inside a cell [14,15,16] according to the following reaction Scheme 1:

RTK belongs to a set of key enzymes catalyzing important cellular processes. These processes include cell cycle control, shape, and movement as well as differentiation, gene transcription, synaptic transmission, and insulin activity [15,16]. It has been estimated that over a third of all proteins in a mammalian cell can be modified by phosphorylation [17]. In conformity with this they are the most intensively studied protein classes in current pharmacological research, as evidenced by the vast number of kinase-targeting agents enrolled in active clinical trials [18]. The whole protein kinase family (also known as the phosphotransferase family) include 90 protein tyrosine kinases, and 43 tyrosine kinase-like proteins. Humans have 58 known RTKs, which fall into 20 subfamilies [19,20]. Due to this fact, it is a major subclass of the human protein kinase and classified into two groups: RTK and non-RTK. On the basis of more than 5000 known crystal structures with or without small molecules it was found that all of them have a similar molecular architecture (Figure 1) [21]. 

Numerous crystal structures have shown that RTK protein organized in two subdomains referred to as: N-lobe also called small lobe and C-lobe also called large lobe. These two lobe regions are connected into a bilobar structure via a flexible region called a hinge (Figure 1) [24] forming an active site cleft apt to bind ATP. The cleft can be triggered into active and inactive mode due to change of conformation of the cleft part called a flexible activation loop (in literature abbreviated as A-loop). The loop is part of RTK starting with a conserved amino acid sequence Asp-Phe-Gly (DFG) and its 3D structure controls access to the hinge region [25,26]. From a structural point of view, the small lobe consists mostly of β sheets while the large lobe consists of α helixes. Activation of tyrosine kinase activates intracellular signaling such as that mediating cell proliferation [23]. In the active state of RTK, the DFG-motif occupies a hydrophobic back pocket adjacent to the ATP binding site. The inactive state is characterized by the DFG-sequence bent out of the hydrophobic back pocket resulting in an activation loop conformation. The A-loop in the latter mode partially blocks the ATP and substrate binding site [24]. Targeting the DFG-out pocket, which is located adjacent to the ATP binding site is frequently referred to as an allosteric pocket, hydrophobic back pocket, or kinase-switch pocket. When an inhibitor binds the receptor in an allosteric way it causes such a conformational change of the ATP binding site that prevents its normal function [27]. RTKs exert function on the transduction of extracellular signals into the cell while the nob-RTKs accomplish intracellular communication. Due to the kinases’ key roles in governing critical cellular processes they have become one of the most important therapeutic targets in pharmacological research for drugs (especially in cancer therapy) over the past two decades [28]. Based on the known X-ray structures of FDA-approved kinase, inhibitors are classified into two classes: irreversible and reversible. Irreversible class inhibitors tend to covalently bind with a reactive nucleophilic residue proximal to the ATP-binding site, resulting in the blockage of the ATP site and irreversible inhibition. Reversible class inhibitors resulting in the blockage of the ATP sites are divided into four main types (assigned as type I-IV) based on the conformation of the binding pocket and the DFG-motif (Figure 1) [19].

## 3. Mode of Action

Abnormal tyrosine phosphorylation is a hallmark of many types of cancer [13,29,30], inflammatory diseases [31,32], and complications of diabetes [33] and other human diseases [18]. At the same time, drugs are being developed that antagonize the responsible protein tyrosine kinases and phosphatases in order to combat these diseases [17]. One such drug is nintedanib, being a nonselective inhibitor of multiple RTK. Due to the fact that RTK acts as surface receptor, it seems understandable that the main action of nintedanib is carried out through the blocking of: (i) the vascular endothelial growth factor (VEGF) (IC_50_ ≈ 13–34 nmol/L); (ii) the platelet-derived growth factor (PDGF) (IC_50_ ≈ 59–65 nmol/L); and (iii) fibroblast growth factor (FGF) (IC_50_ ≈ 37–610 nmol/L) (see Table 1) [21]. These different families of factors have been shown to be potentially involved in pulmonary fibrosis. In accordance with the biochemical data presented as the IC_50_ value gathered in Table 1, it can be seen that nintedanib has a distinctive and narrow range of kinases that are inhibited by nintedanib at pharmacologically relevant concentrations and with the greatest inhibitory effect on VEGF [2].

**Figure 2:**
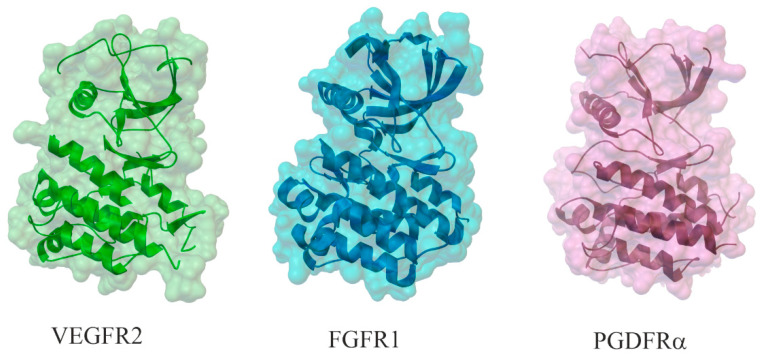
Compression of the selected backbone representation of the crystal structures of the RTK: VEGFR-2 crystal (PDB ID: 3C7Q, 2.10 Å) [21], FGF1 (PDB ID: 1FGK, 2.00 Å) [40], PDGFRα (PDB ID: 6JOL, 1.90 Å) [41].

The drug also affects other non-RTKs, including Src, Flt-3, LCK, LYN [34,37]. Nintedanib blocks tyrosine kinase receptors, including the platelet-derived growth factor receptor (PDGFR), fibroblast growth factor receptor (FGFR), vascular endothelial growth factor receptor (VGFR), by binding to the intracellular ATP binding pocket of the receptor protein domain [38]. In brief, the nintedanib molecule competes with ATP in binding to the receptor pocket, which disrupts dimerization and autophosphorylation of tyrosine kinase domains, as a result it leads to intracellular signaling pathway inhibition [39]. In Figure 2 the backbone representation of the crystal structures of selected receptors is presented.

The VEGF family comprises 4 glycoproteins (VEGFs A-D). VEGF-A and B play a key role in regulating angiogenesis, while VEGF-C and D mainly regulate lymphangiogenesis. The VEGF family binds to VEGFR receptor-1, -2 and -3. Figure 1 exhibits the binding modes between nintedanib and VEGFR-2 receptors confirmed by X-ray data [21]. It can be seen that nintedanib binds inside the ATP-binding pocket in the expected manner. The complex of nintedanib-VEGFR-2 involves an H-bond and ionic interaction. The indolinone moiety of nintedanib forms two hydrogen bonds in the hinge region (with Glu917 and Cys 919) and the 4-nitrogen atom of N-methyl piperazinyl moiety forms a bidentate ionic interaction with the carboxylate oxygens of Glu850 (Figure 1) [21]. It is also known from the crystallographic data that nintedanib binds with the allosteric pocket using its terminal methylcarboxy group forming an H-bond with Leu866 [14]. Additionally, nintedanib has a long terminal chain lying in the outer pocket (i.e., solvent region). According to this data, it classifies as a reversible type II/III inhibitor of VEGFR2. Such molecular activation of the receptor leads to its autophosphorylation and intracellular downstream signaling via Ras, phospholipase C gamma, P38 and PI3K [22]. Blocking the family of VEGF receptors leads to an antiangiogenic effect of the drug [34,42]. Nintedanib blocks the proliferation of three types of cells involved in angiogenesis: endothelium, pericytes and smooth muscles [21]. It has been applied in anticancer treatment. Nintedanib has been approved for the treatment of patients with advanced non-small cell lung cancer. The LUME-Lung 1 and LUME-Lung 2 trials showed an increase in progression-free survival and overall survival in patients with this type of cancer [35]. It is worth emphasizing that in the course of the research it was proven the possibility of blocking kinase activity not only by ATP-binding pocket plugging but also by allosteric binding [14].

The human FGF family consists of 22 proteins, the most important ligands of which are FGF1 and FGF2. There are four types of FGF receptors (FGFR1–4) (Figure 2), each containing three extracellular immunoglobulin-like domains (D1–3) and an intracellular part with a tyrosine kinase function. Ligand binding to the receptor leads to autophosphorylation and activation of the intracellular pathways PI3K, ERK1–2, Ras, Raf, MAPK. This leads to cell proliferation, differentiation and migration [22]. FGF plays an important role in tissue repair, angiogenesis and inflammation. Blocking the FGF/FGFR pathway has antiproliferative, proapoptotic and antiangiogenic effects [36]. It has been shown that blocking FGF receptors reduces alveolar interstitial fibrosis, inhibits proliferation, migration and transformation of fibroblasts into myofibroblasts. Therefore, nintedanib has also been approved as a treatment for idiopathic pulmonary fibrosis [34].

The PDGF family of dimeric proteins consists of PDGF-A, PDGF-B, PDGF-C, PDGF-D, which are four polypeptide chains. These are dimeric ligands that interact with PDGFR alpha and beta homodimers or heterodimers (Figure 2). In the extracellular region of the receptors there are five immunoglobulin-like domains, while the intracellular part has tyrosine kinase properties. As a result of ligand binding, the receptor autophosphorylation occurs, which is followed by signal conduction through the following pathways: Ras, Raf, mitogen-activated protein kinase (MEK), extracellular signal-regulated kinases (ERK), phosphoinositide 3-kinase (PI3K) [22]. The PDGF / PDGFR pathway facilitates cell division, migration and angiogenesis [36]. It is assumed that nintedanib may cause thrombocytopenia and exert anti-inflammatory effects by blocking the PDGFR receptors [34].

Nintedanib has low potential for drug−drug interactions via cytochrome P450 (CYP) 3A4 isoenzyme (IC_50_ > 50 μmol/L), especially with drugs metabolized by CYP [43]. It displays a pH-dependent solubility profile with increased solubility at acidic pH < 3 [39,44]. Nintedanib is a substrate for P-glycoprotein. Strong inhibitors of P-glycoprotein (ketoconazole, erythromycin, cyclosporine) increase the toxicity of nintedanib. Strong inducers of P-glycoprotein (rifampicin, carbamazepine, phenytoin) reduce the body’s exposure to nintedanib [8,9,45,46]

Mechanism of action suggests that nintedanib should be considered as one of the potential agents inhibiting and revising the fibrosis process related to the COVID-19 infections. According to ClinicalTrials.gov there is a clinical trial “Nintedanib for the Treatment of SARS-Cov-2 Induced Pulmonary Fibrosis” (NINTECOR) initiated in October 2020 analyzing clinical possibility of this pharmacological intervention. Because of the known induction of coagulation pathways during COVID-19 infections, the problem of possible interaction between nintedanib and anticoagulant seems to be extremely important.

## 4. Complications

Unfortunately, due to the wide variation in the complications of individual drugs, tyrosine kinase inhibitor drugs, it is not possible to create uniform guidelines for the entire group. Blocking VEGF by nintedanib may lead to decreased platelet activity and leukocyte adhesion, which may increase the risk of bleeding and thrombosis. In turn, blocking PDGF alpha and PDGF beta could lead to thrombocytopenia by affecting thrombocyte production. However, the effect of nintedanib on the coagulation system is low given the fact that nintedanib bioactivity is estimated to be 5% when used at the standard dose of 2 × 150 mg a day for idiopathic pulmonary fibrosis [47]. The most common side effects concerned the gastrointestinal tract, in the form of mild diarrhea, less often nausea or vomiting. In some patients a transient increase in liver enzymes was observed. Although there is a potentially increased risk of bleeding due to VEGFR blockade, no increased incidence of cardiovascular complications or bleeding was observed based on the INPULSIS-1 and INPULSIS-2 trials, despite the fact that nintedanib blocks VEGF and PDGF receptors [48]. In clinical trials, the most common findings were mild epistaxis. There was no significant difference between the study and control groups in the major bleeding rate. However, it should be remembered that patients with an inherited predisposition to bleeding and receiving full-dose anticoagulation were excluded from clinical registration. After the drug had been put on the market there were cases of major bleeding (both in anticoagulant and non-anticoagulant patients). They most often concerned the digestive system, as well as respiratory and CNS bleeding. In turn, arterial thromboembolic events (myocardial infarction, stroke) occurred more frequently in the group using the drug (1.6% vs. 0.5% in INPULSIS). In the case of venous thromboembolism, no increased risk was seen in the subject group (INPULSIS), but there is a potentially increased risk due to the mechanism of action of the drug [47].

### Nintedanib and Bleeding Risk Based on Registration Trials

In INPULSIS on idiopathic pulmonary fibrosis, a slightly higher risk of bleeding was observed in the nintedanib group (*n* = 66, 10.3%) compared to the control group (*n* = 33, 7.8%). The most common bleeding in the study and control group was epistaxis (4.1% in the nintedanib-treated group vs. 0.9% in the control group) and subcutaneous hemorrhage (the nintedanib group: 1.6%, the control group: 0.9%) [49,50]. There were also slight differences in coagulological parameters, namely, prolonged APTT time observed more frequently in the nintedanib group (8.3%) than in the control group (4.7%). However, there were no differences in the INR values. The incidence of major bleeding was similarly low. After the drug introduction, the bleeding rate was similar to that in the INPULSIS trial [47].

In the LUME-Lung trial in patients with non-small cell lung cancer, no significant differences in bleeding adverse events were observed between the study and control groups. Epistaxis was the most common symptom (4.9% in the study group and 2.7% in the control group). Major bleeding was low in both groups (less than 1.5%) and tended to be related to tumor size [47]. In the LUME-Lung trial (patients with adenocarcinoma), the bleeding rate in the study group (nintedanib plus docetaxel) was 10.9% (35 patients out of 320), four of which were severely bleeding. There was no significant difference with the placebo group treated only with docetaxel, where the bleeding rate was 11.1% (37 patients out of 333) and major bleeding occurred in five patients. In the LUME-Lung-1 trial in patients with squamous cell carcinoma of the lung, the bleeding rate was more frequent in the nintedanib group compared to the control group. Patients with recent pulmonary bleeding (>2.5 mL of blood), patients with centrally located tumors and radiographic evidence of large vessel invasion or areas of tumor necrosis were excluded from the study. Prothrombin time was monitored in patients receiving antithrombotic therapy and patients were informed to urgently report any bleeding episodes [51]. There was no correlation between thrombocytopenia and bleeding in the study group. It should be noted that hemorrhagic complications were of a similar, low level despite the use of a higher dose of the drug than in pulmonary fibrosis (200 mg vs. 150 mg) and often in combination with docetaxel (75 mg/m^2^) [52].

In patients who completed the INPULSIS trial, the safety profile was assessed in the INPULSIS-ON extension trial. Despite the continuation of nintedanib treatment, no increased adverse event rate was observed compared to the INPULSIS trial. Diarrhea was the most common adverse event. In the nintedanib group in INPULSIS, who continued the treatment in INPULSIS-ON, the number of bleeding events was 93 events out of 430 patients (event rate: 8.4 per 100 person-years). In the second study group, where patients in INPULSIS received placebo followed by nintedanib in INPULSIS-ON, the number of bleeding events was 49 out of 304 patients (the event rate was 6.7 per 100 person-years). Patients receiving a full dose of anticoagulants or antiplatelet drugs or those requiring fibrinolysis did not participate in the study. Bleeding rate per 100 person-years in patients in INPULSIS was 11.82 in the study group and 8.34 in the placebo group, so extended use of nintedanib in INPULSIS-ON showed that this bleeding rate was even lower than in INPULSIS [53] These results were also confirmed in the analysis of six clinical trials with nintedanib [54].

Postmarketing data showed a similar efficacy and toxicity profile to that seen in clinical trials [55]. In postapproval follow-up studies in the US, the bleeding rate was similar to the rate in INPULSIS (11.9 per 100 person-years) [56].

## 5. Concomitant Use of Nintedanib and Antithrombotic Drugs

Data available from clinical trials on the concomitant use of nintedanib and antithrombotic agents is very limited as this patient group was within the standard exclusion criteria. The studies included patients with low-dose prophylactic heparin, after heparin boluses in intravascular procedures, low-dose ASA (below 325 mg/d), 75 mg/d clopidogrel or an equivalent dose of other drugs. Postmarketing data review showed that over a period of approximately 1 year, bleeding adverse events occurred in less than 5% of 6758 nintedanib-treated patients, with less than 1% of patients having major bleeding events. These data refer to both patients using and not using anticoagulation therapy. The German real-life study concerned 64 patients treated with nintedanib, and 43.7% of them received concomitant anticoagulation therapy: 21.8% received ASA, 10.9% VKA or DOACs and 4.7% combined therapy (ASA + OAC). During a median follow-up of 11 months (1 to 29 months), only 1 bleeding event was noted in the patient receiving combination therapy [57].

### 5.1. New Non-Vitamin K Antagonist Oral Anticoagulants (DOACs−Direct Oral Anticoagulants)

**Figure 3:**
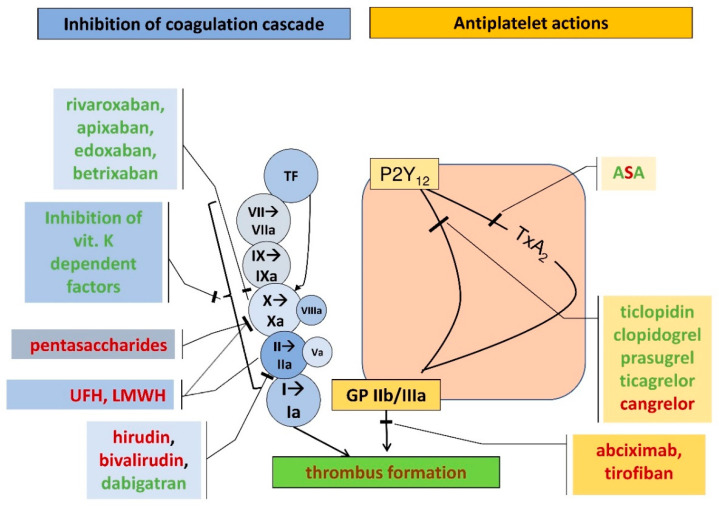
Coagulation cascade (blue rectangles and circles) and the action points of various anticoagulants and antiplatelet agents (yellow rectangles) (UFH, unfractionated heparin; LMWH, low-molecular weight heparin; TF, tissue factor; ASA, acetylsalicylic acid; P2Y_12_, receptor for endogenous nucleotides type 12, TxA_2_, thromboxane A_2_, GP IIb/IIIa, glycoprotein IIb/IIIa). Route of administration: red font—parenteral forms, green font—oral agents.

The search for an ideal anticoagulant drug that can be taken orally at a fixed dose without the need to monitor blood clotting parameters during its use has been ongoing for a long time. Until recently, the only therapeutic option in chronic anticoagulant therapy was the use of oral vitamin K antagonist (VKA) anticoagulants such as warfarin and acenocoumarol. These drugs, despite proven effectiveness in many clinical trials, including in the prevention of embolic complications in patients with atrial fibrillation (AF), have several significant drawbacks that DOACs do not have. The new non-vitamin K antagonist oral anticoagulants have now become a safe and effective alternative to oral vitamin K antagonists. Due to their favorable efficacy and safety profile, predictable anticoagulant effect, no need for constant monitoring of clotting parameters, less intense interactions with food compared to VKA, DOACs have become the drugs of first choice in the anticoagulant treatment of patients with atrial fibrillation [58] (Figure 3).

DOACs fall into two main groups: direct thrombin inhibitors (dabigatran) and Xa inhibitors (rivaroxaban, apixaban and edoxaban) [59]. These drugs inhibit only one clotting factor and do not require any cofactors for activation.

Dabigatran is a potent, competitive, reversible, direct thrombin inhibitor. By inhibiting active thrombin (factor IIa), it prevents the conversion of fibrinogen to fibrin and inhibits free thrombin, fibrin-associated thrombin and fibrin-induced platelet activation. Unlike VKA, it does not affect the synthesis of natural anticoagulants (proteins C and S). In the RE-LY trial, dabigatran at a dose of 150 mg twice daily reduced the incidence of stroke and systemic embolism by 35% compared to warfarin, with no significant difference in the incidence of major bleeding events. On the other hand, dabigatran at a dose of 110 mg twice daily was no worse than warfarin in preventing stroke and systemic embolism and caused 20% fewer episodes of major bleeding [60].

Rivaroxaban is a direct inhibitor of factor Xa which binds both its free and clot-bound form as well as prothrombinase activity. By blocking factor Xa, the thrombin concentration is lowered, which reduces the risk of blood clots forming in the veins and arteries, and helps to dissolve the blood clots that are already present. Rivaroxaban inhibits both the intrinsic and extrinsic coagulation cascades by blocking the enzyme involved in the production of thrombin. The ROCKET-AF trial proved that rivaroxaban at a dose of 20 mg once daily (15 mg once daily in patients with GFR 30–49 mL/min) had a similar efficacy and safety profile as warfarin. The risk of bleeding in both groups was estimated to be 1.03 (95% CI: 0.96−1.11). The incidence of clinically significant bleeding was similar in both groups (rivaroxaban: mean 14.9%, warfarin: mean 14.5%, *p* = 0.44). When analyzing the causes of bleeding events, the risk of intracranial bleeding in the rivaroxaban group was 33% lower, although this beneficial effect was observed in patients <75 years of age. In turn, the incidence of gastrointestinal bleeding was significantly higher in the rivaroxaban group—5.5% (1475/7111 patients) compared to warfarin-treated patients—4.1% (1449/7125 patients) [60,61].

Apixaban is a very selective and potent inhibitor of both free and prothrombinase-bound factor Xa. It does not directly affect platelet aggregation, but indirectly inhibits platelet aggregation induced by thrombin. The specificity of this drug is the presence of numerous elimination pathways, so it does not burden only one organ; it is excreted via the hepatic (75% faeces) and renal (25% urine) routes, and also via the bile and directly through the intestines. Apixaban interacts with inhibitors/inducers of CYP3A4 and P-glycoprotein, and shows little or no interaction with diet and alcohol. The ARISTOTLE trial showed that apixaban compared with warfarin reduced the risk of stroke or systemic embolism by 21%, severe bleeding by 31% and death by 11%. Furthermore, apixaban is the only DOAC that has been compared with acetylsalicylic acid (ASA) in patients with AF. Apixaban was associated with a significant 55% reduction in the incidence of stroke and systemic embolism compared with ASA, with no difference in the incidence of major bleeding and intracranial bleeding [60,61].

Edoxaban is an oral, highly selective, reversible and direct inhibitor of factor Xa—a serine protease found at the end of internal and external coagulation pathways. Inhibiting the activity of this factor leads to a reduction in the amount of thrombin formed, and a prolongation of the clotting time, which reduces the risk of blood clots. Elimination of edoxaban is primarily renal (11 L/h). In patients with renal insufficiency and in people with low body weight (≤60 kg), it is necessary to modify the doses of the drug [62,63]. Edoxaban is also a substrate for the P-glycoprotein (P-gp) transporter, found mainly in the small intestine, responsible for the transport of drugs into the intestinal lumen, which limits their systemic absorption. In the case of concomitant use of potent P-glycoprotein inhibitors, such as quinidine, verapamil or dronedarone, edoxaban doses must be reduced [28,34]. The ENGAGE AF-TIMI 48 trial showed that edoxaban at a dose of 60 mg once daily reduced the incidence of stroke and systemic embolism by 21% compared to warfarin, and edoxaban 30 mg once daily was no worse than warfarin. Major bleeding, intracranial bleeding, and life-threatening bleeding rates were significantly lower in the edoxaban groups compared to those treated with warfarin, with the exception of gastrointestinal bleeding which occurred more frequently in the edoxaban 60 mg group and less often with the dose of 30 mg, compared to warfarin.

### 5.2. Inclusion of DOACs in View of Bleeding Risk

Clinical trials have shown that DOACs are similar in efficacy to vitamin K antagonist oral anticoagulants, but with a lower risk of bleeding [64,65,66,67]. Seven randomized clinical trials showed a reduced risk (32–69%) of major bleeding for dabigatran, rivaroxaban and apixaban compared to VKA [68]. Compared to warfarin, DOACs reduce the risk of intracranial bleeding by an average of 50% [69]. The most ambiguous issue is the effect of DOACs on the gastrointestinal bleeding rate compared to VKA. Research results are often contradictory in this regard. In RE-LY, the use of 2 × 150 mg dabigatran was associated with an increased risk of major gastrointestinal bleeding compared to VKA, and with the dose of 2 × 110 mg the number of bleeding events was comparable to VKA [70]. In another analysis, it was shown that the above risk concerned especially patients over 75 years of age. In the ROCKET-AF trial, rivaroxaban was associated with a more statistically significantly increased risk of major gastrointestinal bleeding, particularly in patients over 75 years of age, than in the VKA group. The ARISTOTLE trial showed a similar rate of major gastrointestinal bleeding in patients receiving apixaban 2 × 5 mg and warfarin [71]. In contrast, the ENGAGE-AF trial showed that the edoxaban dose of 60 mg increased and the dose of 30 mg reduced the risk of serious gastrointestinal bleeding [72].

Based on the conducted trials and the 2016 ESC guidelines for the management of patients with atrial fibrillation, in patients at high risk of gastrointestinal bleeding, the following doses should be considered: VKA or apixaban in the full dose of 2 × 5 mg or other DOACs in reduced doses (dabigatran 2 × 110 mg, rivaroxaban 1 × 15mg, edoxaban 1 × 30mg/d).

### 5.3. Bleeding Risk Scores

The main assumption of anticoagulation therapy is to provide the patient with effective protection against thromboembolic complications and to minimize the risk of bleeding. For this purpose, several scores have been developed, which are aimed at identifying modifiable risk factors for major bleeding. The most recognized scores are:

HAS-BLED score (hypertension, abnormal renal and liver function, stroke, history of bleeding, labile INRs, elderly >65 years of age, drugs/alcohol use) enables the risk of bleeding complications to be assessed and directs attention to potentially reversible risk factors. Special attention should be paid to patients with a HASBLED score of 3 or more, as they should be periodically monitored and potentially reversible risk factors should be corrected [73]. The AMADEUS trial confirmed that the HAS-BLED score best represents the risk of clinically significant bleeding in patients with atrial fibrillation treated with anticoagulants [74].

The ORBIT scale (Outcomes Registry for Better Informed Treatment of Atrial Fibrillation—a registry conducted in 176 centers in America) was created on the basis of a two-year follow-up. The aim of the trial was to identify independent factors influencing serious bleeding complications in patients receiving oral anticoagulants. The researchers created a numerical bedside risk score that included the five risk factors that were easy to measure: (1) age ≥75 years, (2) decreased values of hemoglobin and hematocrit, history of anemia, (3) bleeding history, (4) renal failure, (5) antiplatelet therapy. On this basis, a new tool was created—the so-called ORBIT scale [75].

The latest ESC guidelines for the management of patients with atrial fibrillation recognize the importance of the ABC score: age, biomarkers (GDF-15, hs-TnT, hemoglobin), clinical history of major bleeding. This score was validated in 8468 patients with atrial fibrillation randomized to dabigatran versus warfarin in the RE-LY trial [36]. It was found to reflect major bleeding risk in patients with atrial fibrillation better than the previously used HAS-BLED and ORBIT scores [75,76,77].

According to the ESC guidelines, risk factors for bleeding in patients treated with anticoagulants can be divided into modifiable and nonmodifiable. The first group includes: hypertension, labile INRs or time in the therapeutic range <60% in patients treated with VKA, use of drugs predisposed to bleeding, i.e., anti-aggregating drugs, NSAIDs, excessive alcohol consumption (over 8 drinks a day). Potentially modifiable risk factors for bleeding include: anemia, abnormal kidney function, and abnormal liver function. In turn, the nonmodifiable risk factors for bleeding are: age (>65 years), history of major bleeding, recent stroke, chronic kidney disease requiring dialysis therapy or a transplanted kidney, liver cirrhosis, cancer, genetic factors. There is also a group of bleeding risk factors based on biomarkers such as highly sensitive troponins, growth differentiation factor 15, creatinine clearance or serum creatinine concentration [60].

### 5.4. Antidote

A particularly important consideration when using DOACs is the reversal of drug action in the event of life-threatening bleeding. The first-line specific antidote to dabigatran is idarucizumab, and in its absence, the prothrombin complex factor concentrate (PCC).

Until recently, there was no antidote to reverse the anticoagulant effect of DOACs. However, since 2015, the widely available idarucizumab (an intravenously administered chimeric antibody) can be used as an antidote to dabigatran [78]. It is a fragment of humanized monoclonal antibody that binds to dabigatran with an approximate 300-fold higher affinity than thrombin. The idarucizunab-dabigatran complex has a high binding rate and a very slow degradation rate, which makes it very stable. Idarucizumab binds strongly and specifically to dabigatran and its metabolites, neutralizing their anticoagulant activity. It does not reverse the effects of any other antithrombotic drugs. The recommended dose is 5 g (2 vials of 2.5 mg idarucizumab, 2 boluses or infusions over 5−10 min). No dose adjustment is required in patients with hepatic and renal dysfunction or in the elderly.

The REVERSE-AD trial is a prospective, multicenter, open-label trial that assessed the efficacy and safety of idarucizumab in reversing the anticoagulant effect of dabigatran in patients with uncontrolled bleeding (group A) and for urgent surgery (group B). The trial was conducted in 173 centers in 39 countries and included 503 patients (median age 78 years, 54.5% men) treated with dabigatran in the prevention of stroke in the course of atrial fibrillation (95%), in the primary or secondary prevention of venous thromboembolism (2.4%) and in other indications (2.6%). Idarucizumab was administered in a total dose of 5 g intravenously (2 boluses of 2.5 g each within 15 min apart). The primary endpoint defined as “normalization of the coagulation markers”, used to assess the anticoagulant potency of dabigatran (diluted thrombin time, dTT and ecarin clotting time, ECT) within 4 h of its infusion, occurred in 100% of patients in both groups. Importantly, this effect was independent of the patient’s age, sex, renal function, and trough dabigatran levels. The secondary endpoint defined as restoration of normal hemostasis occurred in 68% of patients within a median of 2.5 h in group A (cessation of bleeding) and in 93% of patients within a median of 1.6 h in group B (normal perioperative hemostasis). Thromboembolic events occurred in 6.3% of group A and 7.4% of group B within 90 days of follow-up. Mortality in both groups was comparable and amounted to 18.8% and 18.9%, respectively, during the 90-day follow-up. Importantly, the frequency of side effects associated with the administration of idarucizumab in the study group was negligible and included one episode of rash, one episode of vomiting and one anaphylactic shock. The results of the REVERSE-AD trial indicated that idarucizumab was an effective and safe antidote to dabigatran in patients who experienced life-threatening bleeding or were indicated for urgent surgery. Based on REVERSE-AD, it can be expected that the safety of dabigatran anticoagulation will increase in centers where idarucizumab is available [78,79,80].

Andexanet alfa is another FXa inhibitor antidote. In May 2018, the drug received accelerated approval from the FDA, while at the beginning of March 2019, the Committee for Medicinal Products of the European Medicines Agency decided to give it a positive opinion [60]. After appropriate modification, andexanet alfa became a recombinant form of the human FXa protein which did not exhibit the enzymatic activity of FXa. Due to the replacement of serine, the active site of FXa, with alanine, the molecule could no longer cleave or activate prothrombin. The gamma-carboxyglutamic acid (Gla) of FXa was also removed to eliminate the protein’s ability to attach to the prothrombinase complex, which led to the disappearance of any anticoagulant effects. Due to its high affinity for FXa inhibitors (rivaroxaban, apixaban and edoxaban), andexanet reversed their effects. Moreover, this drug has the ability to bind to antithrombin activated by low-molecular-weight heparin and fondaparinux. Its main mechanism of action is believed to be binding and sequestration of the FXa inhibitor, although there may be a small contribution to inhibiting the activity of the tissue factor pathway inhibitor (TFPI) through binding to TFPI. So far, the interaction between andexanet alfa and TFPI has not been fully described. Due to the high affinity binding of direct FXa inhibitors by andexanet alfa, they cannot exert antithrombotic effects [81].

Ciraparantag (aripazine, PER977) is a universal agent for factor Xa inhibitors, direct thrombin inhibitors and heparins [78]. It is a small, positively-charged molecule that binds noncovalently to all factor Xa inhibitors (including fondaparinux, low-molecular-weight and unfractionated heparin) and dabigatran, without binding to other clotting factors or albumin. In a study in a mouse tail model, ciraparantag reduced bleeding during treatment with DOACs and normalized clotting time. It had no significant effect on hemostasis when the drug from this group was not used [82]. In healthy volunteers, ciraparantag completely reversed the anti-Xa activity of rivaroxaban and apixaban, even at twice their therapeutic concentration [82]. A placebo-controlled phase I trial on 80 healthy volunteers assessed the safety, tolerability, pharmacokinetic and pharmacodynamic effects of ciraparantag when administered intravenously at doses of 5–300 mg, 3 h after administration of edoxaban 60 mg or alone. Ciraparantag administered at doses of 100−300 mg caused a reversal of the anticoagulant effect within 10−30 min, and its effect was sustained for up to 24 h. Potentially drug-related adverse events included transient facial erythema, dysgeusia, and moderate headache. There were no thromboembolic events associated with the use of ciraparantag and no increase in the concentration of d-dimers and thrombin-antithrombin complexes was observed [83]. The same results were obtained in a trial on healthy volunteers who received a therapeutic dose of enoxaparin [84].

### 5.5. Treatment Monitoring

The advantage of DOACs is that they do not require routine anticoagulation monitoring due to their wide therapeutic index [85]. However, there are circumstances where the actual drug levels may be necessary to know. The material used for DOAC level measurement is usually normal 3.2% (0.109 mol/L) plasma citrate.

An indication for DOAC measurement may be the suspicion of excessively high or low DOAC levels. Lower DOAC levels may occur in obese patients (>110 kg), in the case of treatment failure (recurrent thrombotic episodes) or in malabsorption. Too high DOAC levels can be expected in patients with low body weight (<50 kg), renal failure, bleeding, overdose or in the elderly [86].

The indications for immediate DOAC measurement include injury, urgent surgery or fibrinolytic therapy in acute ischemic stroke [86,87,88]. The presence of DOACs in plasma may be indicated by the prolonged times of routine clotting tests, i.e., prothrombin time (PT) and activated partial thromboplastin time (APTT). However, it should be known that the extended PT or APTT depend on the type of drug and its concentration in the sample. In general, APTT is more sensitive to dabigatran and PT is more sensitive to rivaroxaban, apixaban and edoxaban. However, the PT and APTT reagents demonstrate variable degrees of sensitivity to DOACs. Most PT reagents are less sensitive to apixaban than to rivaroxaban and edoxaban [89,90].

#### 5.5.1. Laboratory Monitoring of Dabigatran Treatment

APTT is affected by dabigatran treatment, but its change depends on the sensitivity of the activator used and the type of coagulometer. In the case of chronic administration of dabigatran at a dose of 150 mg every 12 h, APTT is significantly prolonged (1.5–3 times), but the extent of this prolongation does not closely correlate with dabigatran plasma concentrations, so APTT is not suitable for a precise determination of the degree of anticoagulation. An APTT prolongation exceeding 65–80 s immediately before the next dose of the drug may indicate an excessive reduction in coagulation. APTT and TT values within the normal range indicate no anticoagulant effect of dabigatran. The value of the individual tests in patients treated with dabigatran is as follows:

PT—less sensitive to the anticoagulant effect of dabigatran than APTT; its prolongation is variable and therefore it is not recommended for monitoring the potency of dabigatran; indicative method for assessing the anticoagulant activity of rivaroxaban;

TT—very sensitive to the anticoagulant activity of dabigatran, because even at low concentrations of this drug it becomes undetectable and therefore is not suitable for precise monitoring of its anticoagulant effect;

Diluted thrombin time (dTT = Hemoclot test)—allows for the quantitative measurement of dabigatran plasma concentrations (result expressed in [s] or [ng/ml]); in Europe, the Hemoclot method has been accepted for clinical use and recommended for monitoring the potency of dabigatran.

Ecarin clotting time (ECT)—allows for the quantitative measurement of dabigatran plasma concentrations (the result expressed in [s] are 2- to 4-fold higher in patients who chronically use dabigatran 150 mg every 12 h); the test is recommended for monitoring the potency of dabigatran;

Prolonged activated clotting time (ACT), measured by rapid thromboelastography, can be used for the approximate evaluation of dabigatran effects, and the risk of bleeding in patients taking dabigatran and hospitalized for major injuries. [61,91].

#### 5.5.2. Laboratory Monitoring of Rivaroxaban Treatment

APTT and PT, measured immediately after rivaroxaban administration (especially at higher doses), are prolonged, but the effect is so variable that measuring these parameters is not recommended for monitoring the potency of rivaroxaban. The type of thromboplastin used has a significant influence on the PT measurement result. Fibrinogen and TT levels are not affected by rivaroxaban. The best way to accurately monitor the anticoagulant activity of rivaroxaban is to measure anti-Xa activity. At present work is underway to standardize and validate the test for this indication [92,93].

#### 5.5.3. Laboratory Monitoring of Apixaban Treatment

Apixaban prolongs APTT and PT, however, there is no close correlation of these parameters with the dose and therefore these tests are not recommended for monitoring the potency of apixaban. There is no data on the effect of apixaban on the TT result. As with rivaroxaban, measuring anti-Xa activity is the best way to accurately monitor the anticoagulant activity of apixaban. Work is underway to standardize and validate this test for this indication [94].

A number of medications taken by the patient compete with DOACs for the ATP-dependent transmembrane transporter (P-glycoprotein). This is manifested by the interaction of DOACs with certain drugs, inhibitors or stimulators of this protein. There is little interaction with diet and alcohol, which accounts for the advantage of DOACs over oral vitamin K antagonists. The decision regarding laboratory control of DOAC treatment is an important issue. In field studies, a wide range of therapeutic DOAC levels is observed, from values below 20 ng/mL to above 400 ng/mL [95]. Therefore, there are indications for drug concentration monitoring and individual dose selection, especially in relation to specific groups of patients. Whilst the treatment with DOACs does not require monitoring under standard conditions, there are a number of clinical situations where an assessment of bleeding risk or thrombosis is important [96].

It is possible to measure plasma drug concentrations with the use of traditional chemical methods, e.g., mass spectrometry. Mass spectrometry is considered the “gold standard” for plasma drug concentration measurement due to its precision and accuracy. Unfortunately, this method is not available in most routine hospital laboratories.

### 5.6. Clinical Implications

At present, there are no recommendations regarding the relationship between the use of nintedanib and DOACs. In patients with idiopathic pulmonary fibrosis, there is evidence that VKAs should be used with caution due to their antifibrotic effects. In the IPFnet trial of warfarin in patients with idiopathic pulmonary fibrosis, excess mortality in the drug group resulted in early termination of the trial [97]. Switching from VKA to low-molecular-weight heparin may be considered in patients with mechanical heart valves. There are no clinical studies directly evaluating the use of DOACs in idiopathic pulmonary fibrosis. However, in vitro and in vivo studies suggest some potential benefits, e.g., dabigatran may reduce collagen synthesis, procollagen and fibroblast proliferation by direct inhibition of thrombin [98]. Additionally, inhibition of factor X may reduce lung deposition of collagen in mice and reduce bleomycin-induced pulmonary fibrosis. In AF treatment in patients with pulmonary fibrosis, DOACs have a lower bleeding risk with comparable efficacy to warfarin. However, DOACs increase the risk of gastrointestinal bleeding compared to other anticoagulants, e.g., low-molecular-weight heparins. This is important when deciding whether to use DOACs in combination with nintedanib, which may cause gastrointestinal perforation. Nintedanib should be discontinued in patients requiring surgery until wounds are adequately healed. Based on the mechanism of action nintedanib may impair wound healing [51]. Each patient treated with nintedanib and with indications for concomitant oral anticoagulant use requires an individual assessment of bleeding risk. Such an assessment should be made at the beginning of therapy and then repeated at least once a year. In order to reduce the risk of bleeding, therapeutic drug monitoring (TDM) is worth considering. In practice, TDM means that it is possible to ensure more effective drug action and greater safety in its use. Normally DOACs do not require constant monitoring, however, in the following clinical situations it may be necessary to measure blood levels:

- bleeding during DOAC treatment,
- before surgery or an emergency invasive procedure,
- in patients taking other drugs affecting the pharmacokinetics of DOACs,
- in patients with very low or high body weight,
- in patients taking DOACs with declining renal function,
- perioperative management in patients treated with DOACs,
- reversal of the anticoagulant effect of DOACs,
- suspected overdose of DOACs.

Recently, in many experienced centers TDM has gained great importance and it is very likely that it will soon become commonplace in clinical practice.

## 6. Conclusions

The mechanism of nintedanib action suggests that it should be considered as a potential agent for inhibiting and revising the inflammation-related fibrosis process during idiopathic pulmonary fibrosis as well as COVID-19 infection-related cases. Nintedanib, despite its interaction with anticoagulants, is an important therapeutic option. If anticoagulant therapy is necessary, the more effective and safer option is the concomitant administration of DOACs and nintedanib, especially when drug-monitored therapy will be used in patients at high risk of bleeding complications.

## Figures and Tables

**Table 1:** Nintedanib’s mechanism of action [21,22,34,35,36].

Compound	Protein	Receptor	IC_50_ [nmol/L] [27]
PDGF (platelet-derived growth factor)	A, B, C, D	PDGFR α and β	59 and 65
FGF (fibroblast growth factor)	1, 2	FGFR -1, -2, -3, -4	69, 37, 108, 610
VEGF (vascular endothelial growth factor)	A, B, C, D, E	1, 2, 3	34, 21, 13

IC_50_—measure of the inhibitory nintedanib potency, i.e., half-maximal inhibitory concentration ensuring inhibition of 50% of the functions of the selected receptor.

## Data Availability

No applicable.

## Abbreviations

ACT	Prolonged activated clotting time
AF	Atrial fibrillation
APTT	Activated partial thromboplastin time
ASA	Acetylsalicylic acid
ATC Code	Anatomical therapeutic chemical classification system
ATP	Adenosine triphosphate
COVID-19	Coronavirus disease 2019
DGF	Amino acid sequence Asp-Phe-Gly
DOAC	Direct oral anticoagulants
dTT	Diluted thrombin time
ECM	Extracellular matrix
ECT	Ecarin clotting time
EMA	European Medicines Agency
ERK	Extracellular signal-regulated kinases
FDA	Food and Drug Administration
FGF	Fibroblast growth factor
FGFR	Fibroblast growth factor feceptor
Flt-3	Receptor type tyrosine protein kinase FLT3
GP IIb/IIIa	Glycoprotein IIb/IIIa
HAS-BLED	Bleeding risk in atrial fibrillation score
HAS-BLED	hypertension, age, stroke, prior major bleeding, labile INR, abnormal liver or kidney function, drugs
IC_50_	Half-maximal inhibitory concentration
ICD-10-CM	International Classification of Diseases, Tenth Revision, Clinical Modification
ILD	Interstitial lung diseases
IPF	Idiopathic pulmonary fibrosis
LCK	Lymphocyte-specific protein tyrosine kinase
LMWH	Low-molecular weight heparin
LYN	Tyrosine-protein kinase Lyn
MAPK	Mitogen-activated protein kinase
MEK	Mitogen-activated protein kinase
NSCLC	Non-small cell lung cancer
ORBIT	Outcomes Registry for Better Informed Treatment of Atrial Fibrillation
P2Y12	Receptor for endogenous nucleotides Type 12
PCC	Prothrombin complex factor
PDB ID	Protein Data Bank identification code
PDGF	Platelet derived growth factor
PDGFR	Platelet derived growth factor receptors
P-gl	P-Glycoprotein
PI3K	Phosphoinositide 3-Kinase
PT	Prothrombin time
Raf	RAF kinases, serine/threonine specific protein kinase
Ras	Ras protein, guanosine nucleotide binding protein
RTK	Receptor tyrosine kinases
Src	Src kinase, nonreceptor tyrosine kinase
TDM	Therapeutic drug monitoring
TF	Tissue factor
TFPI	Tissue factor pathway inhibitor
TGA	Therapeutic Goods Administration
TT	Thrombin time
TxA2	Thromboxane A2
UFH	Unfractionated heparin
VEDF	Vascular endothelial growth factor
VEDFR	Vascular endothelial growth factor receptor
VKA	Vitamin K antagonist
